# Endogenous Retroviruses: With Us and against Us

**DOI:** 10.3389/fchem.2017.00023

**Published:** 2017-04-07

**Authors:** Thomas J. Meyer, Jimi L. Rosenkrantz, Lucia Carbone, Shawn L. Chavez

**Affiliations:** ^1^Division of Bioinformatics and Computational Biology, Department of Medical Informatics and Clinical Epidemiology, Oregon Health & Science UniversityPortland, OR, USA; ^2^Department of Molecular and Medical Genetics, Oregon Health & Science UniversityPortland, OR, USA; ^3^Division of Reproductive & Developmental Sciences, Oregon National Primate Research CenterPortland, OR, USA; ^4^Department of Medicine, Knight Cardiovascular Institute, Oregon Health & Science UniversityPortland, OR, USA; ^5^Departments of Obstetrics and Gynecology and Physiology and Pharmacology, Oregon Health & Science University School of MedicinePortland, OR, USA

**Keywords:** endogenous retrovirus, genome, human disease, pre-implantation embryo, stem cells, placenta, innate immunity

## Abstract

Mammalian genomes are scattered with thousands of copies of endogenous retroviruses (ERVs), mobile genetic elements that are relics of ancient retroviral infections. After inserting copies into the germ line of a host, most ERVs accumulate mutations that prevent the normal assembly of infectious viral particles, becoming trapped in host genomes and unable to leave to infect other cells. While most copies of ERVs are inactive, some are transcribed and encode the proteins needed to generate new insertions at novel loci. In some cases, old copies are removed via recombination and other mechanisms. This creates a shifting landscape of ERV copies within host genomes. New insertions can disrupt normal expression of nearby genes via directly inserting into key regulatory elements or by containing regulatory motifs within their sequences. Further, the transcriptional silencing of ERVs via epigenetic modification may result in changes to the epigenetic regulation of adjacent genes. In these ways, ERVs can be potent sources of regulatory disruption as well as genetic innovation. Here, we provide a brief review of the association between ERVs and gene expression, especially as observed in pre-implantation development and placentation. Moreover, we will describe how disruption of the regulated mechanisms of ERVs may impact somatic tissues, mostly in the context of human disease, including cancer, neurodegenerative disorders, and schizophrenia. Lastly, we discuss the recent discovery that some ERVs may have been pressed into the service of their host genomes to aid in the innate immune response to exogenous viral infections.

## Background

**Figure 1:**
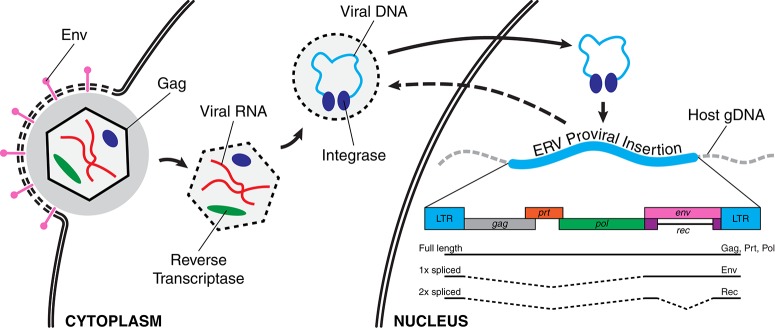
**Retroviral infection and integration into host genome**. Left to right: An infecting viral particle enters the host cell after its envelope, containing Env proteins (pink), fuses with the cell membrane. The viral capsid (hexagon), consisting largely of Gag proteins, contains the RNA form of the retroviral genome (red) as well as a reverse transcriptase (green). The viral genome is subsequently reverse transcribed into its DNA complement (light blue) and this viral genome then enters the nucleus with its associated integrase proteins (dark blue). A new viral integration is then inserted into the host genome, becoming a provirus. Lower right: A schematic of a retroviral genome with components indicated as colored boxes (*gag*, group-specific antigen; *prt*, protease; *pol*, polymerase; *env*, envelope protein; *rec*, accessory protein; LTR, long terminal repeat). Three splice variant transcripts are shown and their translated products given.

**Figure 2:**
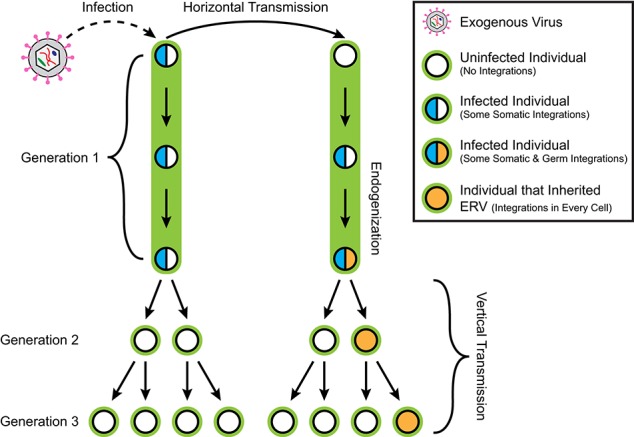
**Retroviral infection, horizontal transmission, endogenization, and vertical transmission**. An exogenous retrovirus infects an individual in generation 1, resulting in their accruing provirus integrations in some somatic cells. Horizontal transmission of the virus from the first individual to the second results in the second accruing somatic integrations as well. However, the second individual subsequently receives germline integrations. The descendants of the first individual do not inherit any retroviral integrations, while any germline integrations in the second individual are transmitted vertically to half of its descendants as endogenous retrovirus insertions present in every cell. Only half of the descendants of this second individual in Generation 2 inherit any given germline integration locus because any cell receiving a new integration does so on only one copy of the affected chromosome. This results in a heterozygous pattern of inheritance.

A retroviral genome exists in different forms during its replication cycle. A viral particle, or virion, protects the RNA genome of the retrovirus during escape from the host cell and infection of new cells. A virion that enters a new host cell deploys its genomic payload, using its own reverse transcriptase to convert the RNA viral genome into a DNA copy which is integrated into the host genome, referred to as a provirus (Figure 1). Subsequently, a provirus can be transcribed into RNA again, and then translated by the host's ribosomal machinery to produce more virions. Ancient retroviral infections have occasionally resulted in such integrations into the germline of the host, becoming endogenous retroviruses (ERVs). While some ERVs have been shown to produce infectious particles (van der Laan et al., 2000), most ERV copies suffer mutations over evolutionary time that prevent the normal assembly of viral particles, preventing horizontal transmission of infections between individuals. However, while now trapped within the host genome, some of these provirus copies are still transcribed and can encode some if not all of the original viral proteins. Therefore, ERVs are classified as a family of autonomous retrotransposons. Further, offspring of the host can inherit any germline ERV insertions from their parents, resulting in a vertical transmission pattern with evolution (Figure 2). As much as 8% of the human genome consists of ERV sequences acquired through repeated endogenization events followed by subsequent retrotranspositional expansion of captured viral subfamilies.

These ancient genomic residents represent a potent source of genomic and regulatory variability. The high degree of homology between these ERV copies, and the presence of the long terminal repeats (LTRs) at either end of each copy (Figure 1), provide an opportunity for non-allelic homologous recombination that can result in the excision of a given insertion, leaving behind only a single LTR copy. Recombination events between the different insertions of the same or similar ERV subfamilies can result in deletions, duplications, and other rearrangements of intervening genomic sequences. Additionally, the ERV sequences themselves can contain motifs that can disrupt or modulate nearby genes and regulatory regions. Not surprisingly, ERVs activity is associated with a number of human diseases and the target of epigenetic repression by the host genome. However, the consequences are not solely deleterious, as there is evidence that ERVs have been co-opted into important regulatory and developmental roles as well.

## ERVs in germ cells and pre-implantation embryos

Certain stages of mammalian pre-implantation embryo and germ cell development characterized by multiple waves of epigenetic reprogramming pose a unique challenge for the control of endogenous retroviral activity. During the two waves of epigenetic reprogramming that occur in primordial germ cells (PGCs) and fertilized oocytes, a considerable amount of DNA demethylation occurs. Examination of global DNA methylation at these stages have shown that levels within human and mouse pre-implantation embryos decrease beginning at the 1- to 2-cell stage, depending on the species, and up to or soon after the blastocyst stage (Kobayashi et al., 2012; Guo et al., 2014; Lee et al., 2014; Okae et al., 2014; Wang L. et al., 2014). Since DNA methylation is largely responsible for repression of many transposable elements, including ERVs (Walsh et al., 1998), the activity of ERVs and the alternative mechanisms repressing ERV activation during these periods of global hypomethylation have been the focus of a number of recent investigations.

Given that some ERV families have expanded substantially in the number of genomic integrations in animals (Tristem, 2000; Bénit et al., 2001), it has been hypothesized that widespread reactivation of ERVs during the waves of global reprogramming within germ cell and pre-implantation development are largely responsible for this expansion. On the other hand, it is also known that additional ERV repressive mechanisms must be in place in order to maintain genomic stability throughout epigenetic reprogramming and the highly choreographed molecular processes required for normal germ cell development, fertilization, and embryonic development. These ideas are not mutually exclusive, as there is substantial evidence supporting both reactivation (Fuchs et al., 2013; Wang J. et al., 2014; Grow et al., 2015) and alternative repression (Thomas and Schneider, 2011; Manghera and Douville, 2013; Leung et al., 2014; Liu et al., 2014; Schlesinger and Goff, 2015; Wolf et al., 2015; Thompson et al., 2016) across the vast number and variety of ERVs within the genome during germ cell development and embryogenesis.

Despite the existence of elaborate mechanisms that mediate ERV inactivation within the genome, there is extensive evidence that some ERVs are still active and play an important role during gametogenesis and pre-implantation development. Upregulation of ERV proviral transcription and protein expression has been well documented in early human embryos and embryonic stem cells (hESCs). For example, elevated expression of the ERV-H family has been observed within both naïve-like and primed hESC sub-populations (Wang J. et al., 2014; Theunissen et al., 2016; Supplementary Table 1). Additional transcripts from the ERV-K (HML-2) family are also observed at high levels within hESCs and rapidly decrease upon differentiation (Fuchs et al., 2013). Expression of ERV-K begins at the 8-cell stage, concurrent with embryonic genome activation (EGA), and continues throughout pre-implantation development into the blastocyst stage. A majority of actively transcribed ERV-K loci during this time are associated with LTR5HS, a specific subclass of LTR, which is confined to human and chimpanzee and contains an OCT4 binding motif. The LTR5HS subclass requires both hypomethylation and OCT4 binding for transcriptional activation, which synergistically facilitated ERV-K expression (Grow et al., 2015; Supplementary Table 1). Based on the elevated activity of these ERVs within hESCs and pre-implantation embryos, as well as their known interactions with other cellular factors during this time, it is thought that these ERVs have been functionally incorporated into roles important for defining and maintaining pluripotent specific states.

The role of LTRs as regulatory regions for proviral DNA represents an additional function that can be utilized by or incorporated into host genomes. In particular, LTRs are known to be co-opted as promoters or enhancer elements of nearby genes important during embryonic development and maintenance of pluripotency (Friedli and Trono, 2015). Nearly, ~33% of all transcripts in human embryonic tissues are associated with repetitive elements, suggesting a clear pattern of embryonic cell specificity for viral promoters (Fort et al., 2014). Many transcripts detected in the totipotent blastomeres of mouse 2-cell embryos are initiated from LTRs upon EGA as well, indicating that these repeat sequences may help drive cell-fate regulation in mammals (MacFarlan et al., 2012). Regulatory activities of certain LTRs have also been shown to provide important functions not only in embryonic cells, but also within germ cells during gametogenesis. For example, germline-specific transactivating p63 (GTAp63), a member of the p53 family and a transcript important for maintaining genetic fidelity in the human male germline, is under the transcriptional control of ERV9 LTR (Ling et al., 2002; Beyer et al., 2011; Liu and Eiden, 2011; Supplementary Table 1). Transcriptionally active GTAp63 suppresses proliferation and induces apoptosis upon DNA damage in healthy testis and is frequently lost in human testicular cancers. Restoration of GTAp63 expression levels in cancer cells was observed upon treatment with a histone deacetylase (HDAC) inhibitor, indicating possible epigenetic control of ERV9-mediated GTAp63 expression via activating histone acetylation marks. Thus, the ability of ERV9 regulatory regions to contribute to the maintenance of male germline stability is yet another example of how ERVs have evolved to serve an important function in their human hosts (Liu and Eiden, 2011).

## ERVs in the placenta

The placenta is a transient organ representing the maternal-fetal interface during pregnancy; it is derived from the outer trophectoderm (TE) layer of blastocysts, and plays a critical role in the gas, nutrient, and waste exchange required for normal embryonic growth. It is well established that both mouse and human placentas are hypomethylated compared to other somatic cells derived from either *in vivo* or *in vitro* sources (Ehrlich et al., 1982; Fuke et al., 2004; Cotton et al., 2009; Popp et al., 2010; Hon et al., 2013). As such, the DNA methylation levels of LTRs within human placentas more closely resemble that observed in oocytes than in somatic tissues, averaging ~60% methylation across the genome (Schroeder et al., 2015). Given this hypomethylation of LTRs in placentas, it is not surprising that numerous sub-families of ERV proviruses are expressed within human placental tissues. More specifically, there is evidence of proviral transcription from ERV-E (Yi and Kim, 2007), ERV3 (ERV-R; Boyd et al., 1993; Andersson et al., 2005), ERV-K (Kammerer et al., 2011), ERV-fb1 (Sugimoto et al., 2013), ERV-V1/2 (Esnault et al., 2013), ERV-W (Blond et al., 2000), and ERV-FRD (Blaise et al., 2003; Supplementary Tables 1, 2).

The most notable ERV families producing functional proteins during placentation are ERV-W and ERV-FRD, corresponding to Syncytin-1 and Syncytin-2, respectively, which are critical for the cellular fusion underlying human placental syncytia formation and maintenance (Blond et al., 2000; Mi et al., 2000; Blaise et al., 2003, 2005; Dunk et al., 2012; Supplementary Table 2). Cellular fusion is a relatively unique function in normal healthy tissues, with muscle, bone and placenta being the major exceptions. Since regulation of this highly specified function is of much interest, the precise mechanisms underlying the transcriptional control of the Syncytin-1 gene have been the topic of several investigations. Both DNA and histone H3K9 methylation have been reported to be important for inactivating ERV-W and thus repressing Syncytin-1 expression, resulting in pathological conditions such as exogenous viral infections and preeclampsia when repression does not occur (Matousková et al., 2006; Gimenez et al., 2009; Li et al., 2014; Zhuang et al., 2014). It has been shown that transcriptional activation of the ERV-W locus and the promotion of cell fusion also requires the synergism of LTR promoter hypomethylation, along with the binding of several transcription factors such as GCM1, Sp1, and GATA family members (Yu et al., 2002; Cheng et al., 2004; Prudhomme et al., 2004; Cheng and Handwerger, 2005; Chang et al., 2011). Recently, another ERV-derived protein called suppressyn has been identified to alternatively regulate Syncytin-1, but not Syncytin-2-based cell fusion by inhibiting its interaction with the Syncytin-1 associated receptor, ASCT2 (Sugimoto et al., 2013; Supplementary Table 2). Suppressyn is a truncation product of the proviral *env* gene from the ERV-fb1 element and is transcribed within the placenta. Within normal human placentas, suppressyn is co-expressed with Syncytin-1 in the syncytiotrophoblast layer (Sugimoto et al., 2013), further supporting that these two factors are involved in cell-cell fusion regulation at the maternal-fetal interface *in utero*.

Notably, integration of ERV-W and ERV-FRD into the genome occurred prior to the divergence of Old World (Catarrhini; Cáceres et al., 2006) and New World (Platyrrhini) monkeys (Blaise et al., 2003), respectively, thus Syncytin-1 and Syncytin-2 are only present in higher-order primate (Haplorhini) species, although functionally similar yet distinct ERV proviral proteins have been discovered throughout most mammalian genomes, as reviewed in Imakawa et al. (2015). The ERV-V *env* gene present within Old World monkeys has also been implicated in trophoblast fusion activity, possibly alleviating the lack of functional Syncytin-1 within these species, while the ERV-V reiterations present within the human genome are not functional in this capacity (Esnault et al., 2013; Supplementary Table 2). Syncytin-A and Syncytin-B appear to function like human Syncytins within the mouse placenta and are known to have entered the murine (Muridae) lineages approximately 20 million years ago (Dupressoir et al., 2005). Similarly, Syncytin-Ory1 has been discovered in rabbits and hares (Leporidae; Heidmann et al., 2009), Syncytin-Car1 within 26 different species of carnivorans (Carnivora; Cornelis et al., 2012), Syncytin-Mar1 within the squirrel-related clade (either Scuridae or Marmotini; Redelsperger et al., 2014), Syncytin-Ten1 within tenrec (Tenrecidae; Cornelis et al., 2014), Syncytin-Rum1 in ruminants (Ruminantia; Cornelis et al., 2013), and Syncytin-Opo1 within the short-lived placenta of opossum and kangaroo marsupials (Marsupialia; Cornelis et al., 2015).

Several ERV captured *env* genes have been proposed to have an immunosuppressive role that is important for preventing maternal rejection of the semi-allogenic fetus during pregnancy. In addition to fusogenic properties derived from the *env* gene of ERV-FRD, Syncytin-2 contains a classical Env retroviral immunosuppressive domain that has been shown to have immunosuppressive activity via *in vitro* tumor-rejection assay (Mangeney et al., 2007). Given observed protein expression within cytotrophoblasts cells of the human placenta, Syncytin-2 has been suggested to facilitate fetal tolerance by suppressing the maternal immune system. Other ERV-derived env proteins from ERV-V and ERV-K have also been proposed to possess an immunosuppressive role in controlling the maternal immune system during pregnancy. This is based on findings that both families have one or more proviral loci in the genome with intact *env* open reading frames (ORFs) and a corresponding immunosuppressive domain. Additionally, both ERV-V and ERV-K expression has been observed within placental trophoblast cells at the maternal-fetal interface, although corresponding *in vitro* functional assays have not yet been completed to directly support *in vivo* findings (Kammerer et al., 2011; Subramanian et al., 2011; Supplementary Table 1). Until these studies are undertaken, the exact function of ERV-V and ERV-K and whether env protein expression from these ERVs induce maternal immunosuppression within the placenta, will remain unknown.

## ERVs and human disease

Through insertional mutagenesis, recombination between homologous copies, and the regulatory disruption that epigenetic suppression of ERV insertions can cause to nearby gene loci, there are many mechanisms by which these elements might cause disease. In particular, their association with various cancers has been well demonstrated, as reviewed in Katoh and Kurata (2013). For instance, ERV activity has been strongly associated with many breast cancers (Golan et al., 2008; Wang-Johanning et al., 2008; Salmons et al., 2014). While in melanoma tissues, ERV-K expression of both RNA and protein has been shown (Büscher et al., 2005), and one recent study identified 24 ERV-K (HML-2) loci transcribed (Schmitt et al., 2013). In another study of Hodgkin's lymphoma, all cancer patient samples were found to have alternative transcripts of the *CSF1R*, an important locus associated with this cancer, that initiate at the LTR of an ERV located ~6.2 kb upstream of the normal promoter (Lamprecht et al., 2010).

ERVs have been demonstrated to be associated with a variety of neurologic diseases, as reviewed in Douville and Nath (2014). One such disease is amyotrophic lateral sclerosis (ALS). Elevated ERV-K (HML-2) activity has been observed in the brain tissue of ALS patients (Douville et al., 2011), while transgenic animals expressing the ERV-K *env* gene in cortical and spinal neurons developed motor dysfunction, suggesting that these elements may contribute to neurodegeneration (Li et al., 2015). Additionally, the expression of ERV-W *env* and *gag* has been observed in samples of muscle from ALS patients (Oluwole et al., 2007). While the ERV-W findings may be due to the inflammatory response (Alfahad and Nath, 2013), the support for the involvement of ERV-K in ALS is mounting, though causality has yet to be demonstrated. Multiple sclerosis (MS) is another neurological disease in which ERVs have been strongly implicated. MSRV (multiple sclerosis-associated retrovirus), a subtype of ERV-W, as well as ERV-W1 and W2 and ERV-H/F have all been linked to MS (reviewed in Christensen, 2016). One study showed significantly elevated Env antigen in serum of MS patients relative to controls, while qPCR of ERV-W in mononuclear cells from blood (PBMC) showed association with MS relative to controls (Perron et al., 2012a). This same study demonstrated Env expression in eight well-characterized MS brains that had lesions throughout the parenchyma and in perivascular infiltrates, as well as at the rim of chronic active lesions. ERV association with schizophrenia and bipolar disorder has been demonstrated through the presence of biomarkers for ERV-K and ERV-W found in blood, cerebrospinal fluid, and the pre-frontal cortex (Karlsson et al., 2001; Huang et al., 2006, 2011; Perron et al., 2012b). In one study of schizophrenia, hypermethylation of a specific ERV-W LTR insertion located in the regulatory region of the *GABBR1* gene was associated with risk of schizophrenia (Hegyi, 2013). A nearly full-length ERV-K insertion near the *PRODH* gene, known to be associated with schizophrenia and other neuropsychiatric disorders, has been shown to work in concert with the internal *PRODH* CpG island to activate the gene. It is thought that aberrant DNA methylation of this locus may be a piece of the schizophrenia puzzle (Suntsova et al., 2013).

## ERVs may play a role in the innate immune response

While the majority of ERV proviruses have acquired mutations, thereby preventing translation into protein, certain families have been especially well preserved and contain functional ORFs for one or more of the classical proviral genes. Within primates, ERV-K (HML-2) represents the best-preserved and most recently active ERV, containing a substantial number of loci that have predicted coding potential throughout different primate genomes. It has also been observed that ERV-K encodes a small accessory protein, Rec, in naïve ES cells and human blastocysts. Overexpression of Rec protein within human pluripotent cells increases the innate antiviral response and can inhibit exogenous viral infections, suggesting an immunoprotective role of the ERV-K Rec protein during early embryonic development (Grow et al., 2015; Supplementary Table 1). An additional ERV-K proviral protein, gag, which makes up the core of viral particles in exogenous retroviruses, is also expressed within human blastocysts and pluripotent cells. Immunolabeling of ERV-K gag protein followed by confocal and transmission electron microscopy revealed ERV-K gag protein within structures of blastocysts resembling viral-like particle (VLPs). This suggested that some ERV proviral sequences within the human genome still retain the ability to code for viral proteins and form VLPs during normal human embryogenesis. Proteins produced from ERV *env* genes have also been demonstrated to function as restriction factors against exogenous retroviral infection (Malfavon-Borja and Feschotte, 2015).

Even ERV proviruses that do not contain functional ORFs can still harbor sequence motifs that serve to modulate the activity of nearby genes. For instance, interferon (IFN)-inducible enhancers have been dispersed via ERV insertions adjacent to IFN-inducible genes independently over mammalian evolution. This has resulted in regulatory networks of genes able to work in concert due to the presence of these ERV sequences. Further, CRISPR-Cas9 deletion of a MER41 insertion upstream of *AIM2* in HeLa cells disrupted the endogenous IFNG-inducible regulation of this locus, demonstrating the utility that host genomes can obtain over time by harnessing ERV sequences (Chuong et al., 2016). In another example showing the variety of mechanisms by which ERVs are involved with innate immunity, Chiappinelli et al. (2015) demonstrated that induction of ERV expression, and especially bidirectional transcription of ERVs, activated a double-stranded RNA sensing pathway that triggers a type I interferon response and apoptosis.

## Conclusions

The relationship between ERVs and the human genome is a diverse and complicated one, resulting from millions of years of co-evolution. ERVs are known to be involved in disease through insertional mutagenesis, as targets of epigenetic repression, and via recombination of sequences between the homologous copies of these elements scattered across the genome. Throughout mammalian evolution, the deleterious effects of ERVs have been balanced by the benefits gained from innovative co-option of their sequences and proteins by their host genomes. These innovations include the intimate relationship between ERV activity with embryonic and placental development, as well as a number of ERV-associated regulatory networks that have become important components of the normal function of our genome. An innate immune response to exogenous retroviral infection is likely only one of several ERV functional roles. Once thought to have been quiescent, dead residents of the human genome, we are only beginning to uncover the scope of how actively intertwined our biology is with these long-time genomic partners.

## Author contributions

TM and JR drafted the manuscript and figures. SC and LC edited the manuscript.

## Funding

TM was supported by the National Library of Medicine of the National Institutes of Health under Award Number T15LM007088. JR was supported by the Collins Medical Trust Foundation and Glenn/AFAR Scholarship for Research in the Biology of Aging. LC and SC were supported by NIH/NICHD R01HD086073-A1, National Centers for Translational Research in Reproduction and Infertility (NCTRI). Additional funding for SC came from the Georgeanna Jones Foundation for Reproductive Medicine, Medical Research Foundation of Oregon, and the Collins Medical Trust. The content is solely the responsibility of the authors and does not necessarily represent the official views of the National Institutes of Health, the Collins Medical Trust Foundation, or Medical Research Foundation of Oregon.

### Conflict of interest statement

The authors declare that the research was conducted in the absence of any commercial or financial relationships that could be construed as a potential conflict of interest.
