# Supplementary material for: Endogenous Retroviruses: With Us and against Us
Source: Front Chem. 2017 Apr 7;5:23. doi: 10.3389/fchem.2017.00023 (PMC5384584; doi:10.3389/fchem.2017.00023)
Supplement: Supplementary file 1 [file Tables1and2.docx]

Supplementary Material

Endogenous Retroviruses: With Us and Against Us

Thomas J. Meyer, Jimi L. Rosenkrantz, Shawn L. Chavez, Lucia Carbone^*^

*** Correspondence:**

Thomas J. Meyer

thomas.joshua.meyer@gmail.com

# Supplementary Tables

**Supplementary Table 1: Functional ERVs during early development & placentation that are widely distributed in the genome.** Key: CT cytotrophoblast; EVT extravillous trophoblast; ST syncytiotrophoblast; VLP virus-like particle; ~ no specific gene.

| **Family** | **Gene** | **Function** | **References** |
| --- | --- | --- | --- |
| ERV-H | ~ | Marker of pluripotent hESC; important component of primate pluripotent transcriptional profile. | Wang et al. 2014a |
| ERV-K (HML2) | *rec* | Overexpression of REC increases innate antiviral response and can inhibit exogenous viral infections within human pluripotent cells. | Grow et al. 2015 |
|  | *gag* | GAG protein is expressed and contributes to VLP formation in human blastocyst stage embryos. | Grow et al. 2015 |
|  | *env* | ENV is expressed within human placental CT and EVT cells and postulated to play a role in cell fusion and/or immunosuppression. | Kammerer et al. 2011 |
|  | LTR5hs | OCT4 binding motif increases transcriptional control of associated ERV-K elements. | Grow et al. 2015 |
| ERV9 | ERV9-LTR | U3 region of the LTRs possesses strong enhancer activity in embryonic cells of widely different tissue origins. E.g. Transcriptional control of GTAp63, important for maintaining genetic fidelity in the human male germ line. | Ling et al. 2002; Beyer et al. 2011; Liu and Eiden 2011 |
| ERV-E | *env*-containing | ERV-E *env* proteins have been detected in placenta, where they may affect normal cell fusion. Additionally, the ERV-E family has been shown to participate in the regulation of several human genes by providing a tissue-specific enhancer several genes, and is also involved in transcriptional regulation of human genes by contributing an alternative promoter to genes. | Yi and Kim 2007 |

**Supplementary Table 2: Functional ERVs during early development & placentation that have unique map locations.** Key: CT cytotrophoblast; EVT extravillous trophoblast; ST syncytiotrophoblast.

| **Family** | **ERV Locus** | **Gene** | **Chromosome** | **Expression/Function** | **References** |
| --- | --- | --- | --- | --- | --- |
| ERV-W | ERV-W-1 | Syncytin-1 (*env*) | 7q21.2 | Expressed within CT and ST of human placenta; important for cell-cell fusion during placentation. | Mi et al. 2000; Blond et al. 2000; Dunk et al. 2012 |
| ERV-FRD | ERV-FRD | Syncytin-2 (*env*) | 6p24.1 | Expressed within CT of human placenta; putative ISU domain and role; important for cell-cell fusion during placentation | Blaise et al. 2003; Blaise et al. 2005 |
| ERV-Fb | ERV-Fb1 | Suppressyn (*env*) | 21q22.3 | Expressed within ST and EVT of human placenta; it binds to syn-1 receptor and inhibits syn-1 mediated cell-cell fusion. | Sugimoto et al. 2013 |
| ERV-V | ERV-V2 | *EnvV2* | 19q13.41 | Not fusogenic in humans, although has retained fusogenic activity in Old World monkeys as well as some New World monkey species, possibly alleviating the lack of functional syncytin-1 within these species. | Esnault et al. 2013 |
| ERV3 (ERV-R) | ERV3-1 | *env-*containing | 7q11.2 | ERV3-1 is highly expressed in a number of human cancers, as well as healthy epithelial cells, immune cells, liver, heart, placenta trophoblasts and stomach chief cells. Highly expressed within tissues related to reproduction and endocrine functions. Promotes cell fusion when expressed within BeWo trophoblastic cell line. | Boyd et al. 1993; Andersson et al. 2005 |
